# Supplementary material for: Structural characterization of human Vaccinia-Related Kinases (VRK) bound to small-molecule inhibitors identifies different P-loop conformations
Source: Sci Rep. 2017 Aug 8;7:7501. doi: 10.1038/s41598-017-07755-y (PMC5548783; doi:10.1038/s41598-017-07755-y)
Supplement: Supplementary file 1 — Supplementary Information [file 41598_2017_7755_MOESM1_ESM.pdf]

Structural characterization of human Vaccinia-Related Kinases (VRK) bound to small-molecule inhibitors identifies different P-loop conformations

Rafael M. Couñago[1,2*], Charles K. Allerston[3], Pavel Savitsky[3], Hatylas Azevedo[4], Paulo H Godoi[1,5], Carrow I. Wells[6], Alessandra Mascarello[4], Fernando H. de Souza Gama[4], Katlin B. Massirer[1,2], William J. Zuercher[6], Cristiano R.W. Guimarães[4] and Opher Gileadi[1,3]

1. Structural Genomics Consortium, Universidade Estadual de Campinas — UNICAMP, Campinas, SP, Brazil.
2. Centro de Biologia Molecular e Engenharia Genética, Universidade Estadual de Campinas, Campinas, SP, Brazil.
3. Structural Genomics Consortium and Target Discovery Institute, Nuffield Department of Clinical Medicine, University of Oxford, UK.
4. Aché Laboratórios Farmacêuticos SA, Guarulhos, SP, Brazil.
5. Department of Biochemistry and Tissue Biology, Institute of Biology, State University of Campinas, Campinas, Brazil.
6. Structural Genomics Consortium, UNC Eshelman School of Pharmacy, University of North Carolina at Chapel Hill, NC, USA.
*Correspondence to Rafael M. Couñago: rafael.counago@unicamp.br (RMC)

Supplementary information

SUPPLEMENTARY METHODS

PKIS results analyses - hierarchical cluster analysis (HCL)

A hierarchical clustering (HCL) analysis was performed to group kinases based on their inhibition patterns across the compounds. The average distance clustering method was employed, using sample tree selection and sample leaf order optimization. The distance metric used was the Pearson correlation and the HCL analysis was performed in the TmeV software [1].

SUPPLEMENTARY REFERENCES

1 Saeed, A. I. *et al.* TM4: a free, open-source system for microarray data management and analysis. *BioTechniques* **34**, 374-378, (2003).

SUPPLEMENTARY FIGURES LEGENDS

Supplementary Figure S1: Hierarchical clustering analysis of PKIS data. Hierarchical clustering analysis of PKIS data. VRK1 clusters with other kinases such as TNIK and CDK2, indicated with stars. Kinases were clustered according to their thermal shift values obtained in the DSF experiments. The distance metric used was the Pearson correlation. Each leaf in the tree represents one kinase and the VRK1 cluster is highlighted with pink leaves. The blue color in each lane corresponds to the thermal shift values.

Supplementary Figure S2: Assembly of hydrophobic spines in active VRKs. Organization of conserved hydrophobic residues (shown as spheres) in the so-called spine of (**a**)VRK1$_{3\text{-}364}$/ASC24 (chain A, green cartoon) and (**b**) VRK214-335/BI-D1870 (pink cartoon). For VRK1$_{3\text{-}364}$/ASC24, location of the surface reduction mutation (SER) clusters 1 to 3 are shown as spheres (Cαs), the fourth SER cluster locates to the disordered C-terminal region and was not included in the final model.

Supplementary Figure S3: Details of the P-loop region in VRK1$_{3\text{-}364}$-GW297361X co-crystals and apo-VRK1 NMR ensemble in solution. All 20 members of the NMR ensemble were superposed to the C-terminal domain (residues 141-341) of VRK1$_{3\text{-}364}$ bound to GW297361X. NMR structures are shown as grey ribbons, except for the C-terminal regulatory domain, in blue, and the P-loop region, in pink. The co-crystal structure of VRK1$_{3\text{-}364}$ / GW297361X is shown as a cartoon model in green, except for the P-loop region, in red. Residue Phe48 from the P-loop and ligand GW297361X are shown in stick representation. The Cα atoms for residue Phe48 in the co-crystal structure and in the two of the NMR models are shown as spheres. Cα atoms for residues within the DYG motif are shown as spheres.

Supplementary Figure S4: Crystal contacts in the P-loop region of VRK1 and VRK2. (**a**) No other atom is within a 4.5 Å radius of the folded P-loop (chain A) in VRK1$_{3\text{-}364}$/GW297361X crystals. Chain A is shown as a green cartoon, the other

VRK1 molecules in the crystal asymmetric unit (chains B-D) are shown as green ribbons. Cα trace for symmetry-related molecules are shown as white ribbons. The ligand is shown in stick representation. (**b**) VRK2$_{14\text{-}335}$/BI-D1870 crystals have a single protein-ligand complex in the asymmetric unit (pink cartoon). The only identified atoms within a 4.5 Å radius of the folded P-loop region in VRK2 are those from the side chain of Pro319 from a symmetry-related molecule (indicated by a prime) to P-loop residue Ser37.

SUPPLEMENTARY FIGURES

Supplementary Figure S1

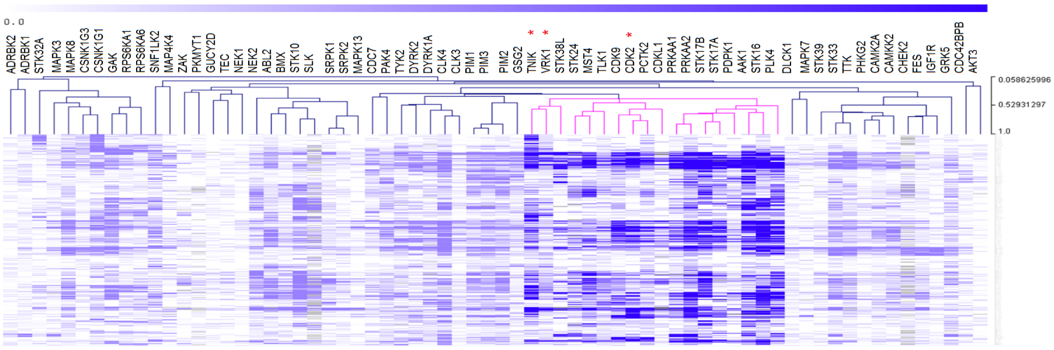

Supplementary Figure S2

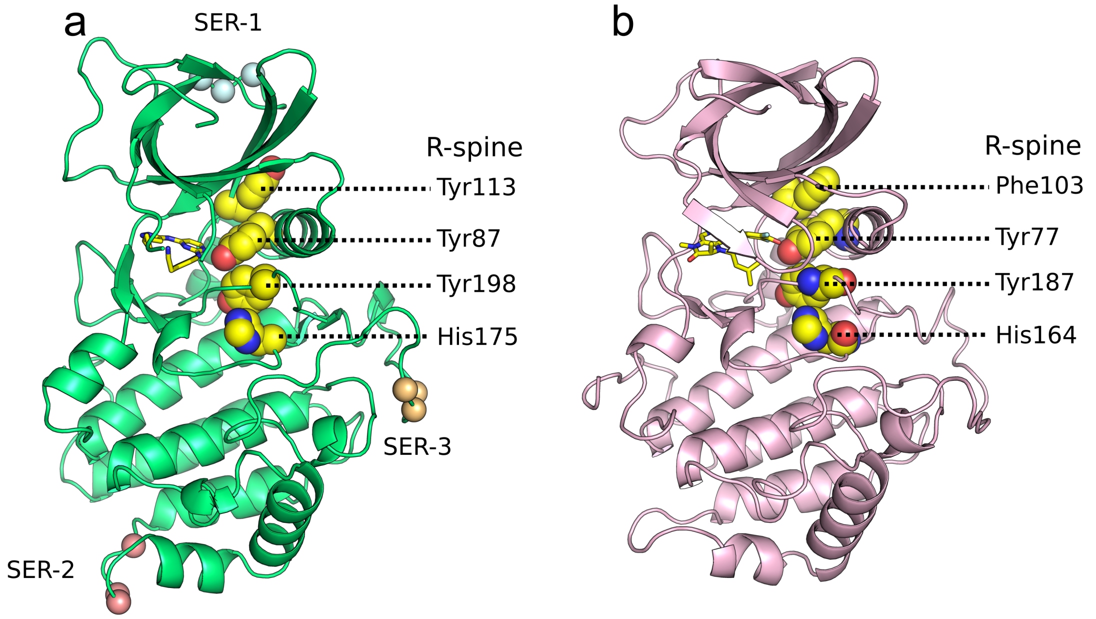

Supplementary Figure S3

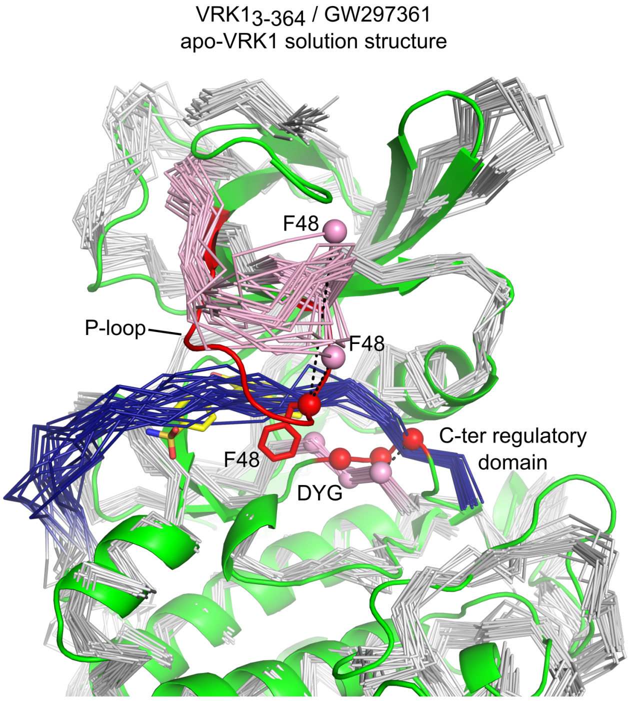

Supplementary Figure S4

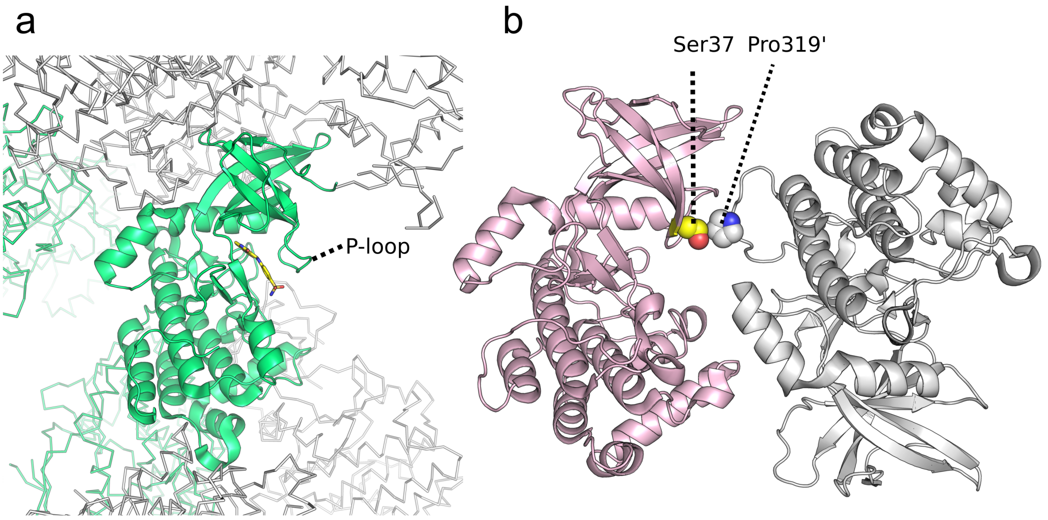

VRK1$_{3\text{-}364}$/GW297361X VRK2$_{14\text{-}335}$/BI-D1870

SUPPLEMENTARY TABLES

Supplementary Table S1

Supplementary Table S1 - VRK1 DSF PKIS hits (68 kinases tested)			
Compound (PKIS number)	Chemotype (PKIS number)	VRK1 ΔTm (°C)	Number of kinases with ΔTm > 2.0°C (rank of VRK1)
GW297361X	02:Oxindoles	9.7	46 (10)
GW589933X	02:Oxindoles	8.0	50 (13)
GW770249A	18:Furopyrimidines_and_related	7.4	31 (8)
GW405841X	02:Oxindoles	6.3	30 (11)
GW290597X	02:Oxindoles	6.0	22 (7)
GW406108X	02:Oxindoles	5.9	32 (16)
GW770249X	18:Furopyrimidines_and_related	4.9	34 (13)
GW300660X	02:Oxindoles	4.8	26 (16)
GW305178X	02:Oxindoles	4.7	31 (18)
GW795486X	18:Furopyrimidines_and_related	4.5	21 (11)
GW429374A	02:Oxindoles	4.3	27 (19)
GW300657X	02:Oxindoles	4.2	28 (13)
GW831091X	27:3-amino_pyrazoles	3.6	22 (10)
GW416981X	02:Oxindoles	3.6	45 (34)
GW407323A	02:Oxindoles	3.5	24 (12)
GW711782X	06:4-pyridyl_ortho-aryl_azoles	3.1	9 (4)
GW795493X	18:Furopyrimidines_and_related	3.0	34 (24)
GW779439X	07:2H-3_pyrimidinyl_pyrazolopyridazines	2.9	51 (47)
GW305074X	02:Oxindoles	2.8	25 (20)
GW695874X	06:4-pyridyl_ortho-aryl_azoles	2.8	6 (4)
SB-236687	01:4-pyrimidinyl_ortho-aryl_azoles	2.7	7 (7)
GSK312948A	05:Benzimidazole_N-thiophenes	2.7	17 (10)
GW806742X	16:2,4-diamino_pyrimidines	2.4	37 (35)
GW837331X	28:Imidazotriazine	2.3	28 (23)
GW852849X	05:Benzimidazole_N-thiophenes	2.1	15 (14)
GW280670X	02:Oxindoles	2.1	26 (26)
GW829055X	2-aryl_3-pyridimidinyl_pyrazolopyridazines	2.0	20 (20)

Supplementary table S2

Supplementary Table S2: Selleckchem Kinase Inhibitor set					
Well	Compound	M.W. (Da)	Known Targets	ΔTm (°C)	
				VRK1	VRK2
A01	Linifanib (ABT-869)	375.41	CSF-1R,PDGFR,VEGFR	-0.14	0.31
A02	ENMD-2076	375.47	Aurora Kinase,FLT3,VEGFR	0.31	0.26
A03	Cediranib (AZD2171)	450.51	VEGFR	-0.24	0.07
A04	Amuvatinib (MP-470)	447.51	FLT3,c-RET,PDGFR,c-Kit	#N/D	#N/D
A05	Lapatinib (GW-572016) Ditosylate	925.46	HER2,EGFR	0.68	0.03
A06	TG100-115	346.34	PI3K	-0.05	0.43
A07	Sorafenib Tosylate	637.03	PDGFR,Raf,VEGFR	#N/D	0.48
A08	PHA-680632	501.62	Aurora Kinase	0.13	0.40
A09	AC480 (BMS-599626)	567.01	HER2,EGFR	-0.71	0.19
A10	HMN-214	424.47	PLK	-0.05	0.25
A11	SB203580	377.43	p38 MAPK	-0.22	0.58
A12	AT7519	382.24	CDK	-0.03	0.22
A13	KU-55933 (ATM Kinase Inhibitor)	395.49	ATM/ATR	-0.45	-0.14
A14	AZD8055	465.54	mTOR	0.00	0.10
A15	LY294002	307.34	Autophagy,PI3K	-0.27	0.03
A16	Fasudil (HA-1077) HCl	327.83	ROCK,Autophagy	-0.35	0.74
A17	Palbociclib (PD-0332991) HCl	483.99	CDK	-0.40	#N/D
A18	AZD8330	461.23	MEK	-0.19	0.09
A19	Brivanib Alaninate (BMS-582664)	441.46	VEGFR,FGFR	-0.31	#N/D
A20	GSK461364	543.60	PLK	-0.15	-0.04
A21	CP-724714	469.53	EGFR,HER2	-0.32	0.18
A22	Mubritinib (TAK 165)	468.47	HER2	-0.13	0.09
A23	JNK Inhibitor IX	332.42	JNK	-1.29	-0.07
A24	Tyrphostin AG 1296	266.29	FGFR,c-Kit,PDGFR	-0.50	0.82
B01	Axitinib	386.47	c-Kit,VEGFR,PDGFR	-0.11	-0.18
B02	CUDC-101	434.49	HDAC,HER2,EGFR	-0.25	0.00
B03	Dovitinib (TKI-258, CHIR-258)	392.43	FGFR,FLT3,c-Kit,VEGFR,PDGFR	0.33	0.67
B04	JNJ-7706621	394.36	CDK,Aurora Kinase	0.49	0.28
B05	Motesanib Diphosphate (AMG-706)	569.44	VEGFR,PDGFR,c-Kit	-0.23	-0.22
B06	GSK1059615	333.36	PI3K,mTOR	0.43	0.71
B07	Sunitinib Malate	532.56	VEGFR,PDGFR,c-Kit	-0.10	0.37
B08	VX-745	436.26	p38 MAPK	-0.37	-0.12
B09	Masitinib (AB1010)	498.64	PDGFR,c-Kit	-0.02	0.40
B10	AEE788 (NVP-AEE788)	440.58	HER2,VEGFR,EGFR	-0.21	-0.10
B11	SB202190 (FHPI)	331.34	p38 MAPK	#N/D	0.70
B12	Quizartinib (AC220)	560.67	FLT3	-1.17	#N/D
B13	GSK1904529A	851.96	IGF-1R	-0.26	0.06
B14	PHT-427	409.61	PDK-1,Akt	-0.74	-1.10
B15	OSU-03012 (AR-12)	460.45	PDK-1	#N/D	0.16
B16	BIRB 796 (Doramapimod)	527.66	p38 MAPK	-0.13	0.10

B17	Triciribine	320.30	Akt	-0.03	#N/D
B18	KW-2449	332.40	Aurora Kinase,Bcr-Abl,FLT3	-0.15	0.45
B19	AG-490 (Tyrphostin B42)	294.30	JAK,EGFR	-0.16	0.65
B20	R406	628.63	Syk,FLT3	-0.57	0.07
B21	TGX-221	364.44	PI3K	-0.09	0.36
B22	PP242	308.34	mTOR,Autophagy	0.35	0.45
B23	PQ 401	341.79	IGF-1R	0.16	-0.02
B24	P276-00	438.30	CDK	0.08	0.47
C01	Saracatinib (AZD0530)	542.03	Src,Bcr-Abl	-0.04	-0.35
C02	PIK-75	488.74	PI3K,DNA-PK	4.35	4.18
C03	PD184352 (CI-1040)	478.67	MEK	-0.08	-0.54
C04	PD173074	523.67	VEGFR,FGFR	-0.35	0.09
C05	Nilotinib (AMN-107)	529.52	Bcr-Abl	0.95	-0.19
C06	MGCD-265	517.60	Tie-2,VEGFR,c-Met	-3.37	-1.67
C07	Tandutinib (MLN518)	562.70	FLT3	-0.24	0.08
C08	Thiazovivin	311.36	ROCK	-0.22	0.15
C09	GDC-0941	513.64	PI3K	-0.03	0.01
C10	PHA-793887	361.48	CDK	0.36	1.27
C11	MK-2206 2HCl	480.39	Akt	-0.02	0.14
C12	Hesperadin	516.65	Aurora Kinase	0.10	1.50
C13	PF-04217903	372.38	c-Met	-0.13	-0.22
C14	KRN 633	416.86	PDGFR,VEGFR	-0.30	0.13
C15	Danusertib (PHA-739358)	474.55	c-RET,FGFR,Bcr-Abl,Aurora Kinase	0.35	0.62
C16	Tie2 kinase inhibitor	439.53	Tie-2	-0.15	-0.31
C17	XL147	448.52	PI3K	0.21	-0.11
C18	RAF265 (CHIR-265)	518.41	VEGFR,Raf	1.77	-2.81
C19	SNS-032 (BMS-387032)	380.53	CDK	-0.17	0.31
C20	SGI-1776 free base	405.42	Pim	0.04	0.72
C21	WZ3146	464.95	EGFR	-0.39	0.11
C22	CYT387	414.46	JAK	-0.15	-0.09
C23	XMD8-92	474.55	ERK	-0.36	0.23
C24	NSC 23766	530.96	Rac	-0.18	0.14
D01	Selumetinib (AZD6244)	457.68	MEK	-0.07	-0.08
D02	Tivozanib (AV-951)	454.86	VEGFR,PDGFR,c-Kit	0.34	-0.44
D03	Dasatinib	488.01	Bcr-Abl,c-Kit,Src	-0.19	0.80
D04	WYE-354	495.53	mTOR	-2.91	-0.50
D05	NVP-AEW541	439.55	IGF-1R	-0.23	0.31
D06	Rigosertib (ON-01910)	473.47	PLK	-0.17	0.02
D07	Temsirolimus (CCI-779, NSC 683864)	1030.29	mTOR	-0.43	-0.75
D08	SP600125	220.23	JNK	0.73	1.86
D09	SL-327	335.35	MEK	-0.23	-0.04
D10	PIK-93	389.88	PI3K	-0.07	-0.11
D11	SU11274	568.09	c-Met	-0.03	-0.37
D12	BIX 02188	412.48	MEK	0.64	0.29
D13	MLN8054	476.86	Aurora Kinase	-0.20	-0.40
D14	AT7867	337.85	S6 Kinase,Akt	0.13	-0.09

D15	BI 2536	521.66	PLK	-0.08	0.07
D16	H 89 2HCl	519.28	PKA	-0.16	-0.05
D17	Cabozantinib (XL184, BMS-907351)	501.51	FLT3,Tie-2,c-Kit,c-Met,VEGFR,Axl	0.05	0.37
D18	AZD1480	348.77	JAK	-0.12	0.36
D19	Barasertib (AZD1152-HQPA)	507.56	Aurora Kinase	-0.04	0.11
D20	BMS-794833	468.84	VEGFR,c-Met	1.11	3.30
D21	CYC116	368.46	Aurora Kinase,VEGFR	0.08	0.17
D22	SB590885	453.54	Raf	-0.24	-0.22
D23	ZM 39923 HCl	367.91	JAK	-1.00	-6.08
D24	Bardoxolone Methyl	505.69	IκB/IKK	-1.09	-2.72
E01	Nintedanib (BIBF 1120)	539.62	VEGFR,PDGFR,FGFR	-0.13	0.04
E02	YM201636	467.48	PI3K	-0.16	-0.49
E03	Ridaforolimus (Deforolimus, MK-8669)	990.21	mTOR	-0.43	-0.51
E04	Vemurafenib (PLX4032, RG7204)	489.92	Raf	#N/D	1.32
E05	Pazopanib HCl (GW786034 HCl)	473.98	VEGFR,PDGFR,c-Kit	-0.34	-0.01
E06	Ki8751	469.41	PDGFR,c-Kit,VEGFR	1.14	0.41
E07	Vandetanib (ZD6474)	475.35	VEGFR	-0.43	-0.23
E08	AZD6482	408.45	PI3K	-0.18	-0.28
E09	Crizotinib (PF-02341066)	450.34	c-Met,ALK	-0.35	-0.49
E10	Ponatinib (AP24534)	532.56	PDGFR,FGFR,VEGFR,Bcr-Abl	-0.54	-0.80
E11	Brivanib (BMS-540215)	370.38	FGFR,VEGFR	-0.09	-0.84
E12	BIX 02189	440.54	MEK	-0.12	-0.21
E13	Vatalanib (PTK787) 2HCl	419.73	c-Kit,VEGFR	-0.52	-0.37
E14	BMS-777607	512.89	Axl,c-Met	-0.21	-0.30
E15	Foretinib (GSK1363089)	632.65	VEGFR,c-Met	0.15	-0.33
E16	TWS119	318.33	GSK-3	0.02	-0.27
E17	Everolimus (RAD001)	958.22	mTOR	-0.68	-1.02
E18	PF-4708671	390.41	S6 Kinase	-0.31	0.38
E19	PLX-4720	413.83	Raf	-0.38	-0.58
E20	NVP-BHG712	503.48	Raf,Src,Bcr-Abl,VEGFR,Ephrin receptor	0.93	-0.19
E21	WZ4002	494.18	EGFR	-0.55	-0.32
E22	Apatinib	493.58	VEGFR	-0.37	-0.01
E23	GNF-5	418.37	Bcr-Abl	-0.05	-0.34
E24	PRT062607 (P505-15, BIIB057) HCl	429.91	Syk	0.01	0.94
F01	Afatinib (BIBW2992)	485.94	EGFR,HER2	-0.28	-0.20
F02	OSI-930	443.44	c-Kit,CSF-1R,VEGFR	1.11	-0.38
F03	Erlotinib HCl (OSI-744)	429.90	Autophagy,EGFR	-0.15	-0.38
F04	BX-795	591.47	IκB/IKK,PDK-1	0.16	0.70
F05	PD0325901	482.19	MEK	-0.21	-0.21
F06	Ruxolitinib (INCB018424)	306.37	JAK	-0.04	0.67
F07	VX-680 (Tozasertib, MK-0457)	464.59	Aurora Kinase	0.53	-0.64
F08	TSU-68 (SU6668, Orantinib)	310.35	VEGFR,PDGFR,FGFR	-0.07	1.41
F09	PHA-665752	641.61	c-Met	-0.60	-0.58
F10	LY2228820	612.74	p38 MAPK	0.96	2.65
F11	NVP-ADW742	453.58	IGF-1R	-0.14	0.06
F12	AZD7762	362.42	Chk	-0.02	-0.10

F13	U0126-EtOH	426.56	MEK	-0.03	-0.20
F14	PD318088	561.09	MEK	0.07	-0.83
F15	SGX-523	359.41	c-Met	-0.04	-0.30
F16	Acadesine	258.23	AMPK	0.03	-0.22
F17	BMS-754807	461.49	IGF-1R,Trk receptor,c-Met	-0.35	-0.50
F18	LY2784544	469.94	JAK	-0.54	-0.04
F19	Roscovitine (Seliciclib,CYC202)	354.45	CDK	-0.45	1.95
F20	OSI-420	415.87	EGFR	-0.19	-0.15
F21	PD98059	267.28	MEK	-0.37	-0.26
F22	CAL-101 (Idelalisib, GS-1101)	415.42	PI3K	-0.43	-0.11
F23	SMI-4a	273.23	Pim	-0.65	-0.71
F24	DMSO	78.13	reference	-0.09	-0.10
G01	BMS-536924	479.96	IGF-1R	0.83	0.69
G02	KU-0063794	465.54	mTOR	-0.09	-0.46
G03	Gefitinib (ZD1839)	446.90	EGFR	-0.36	-0.23
G04	BX-912	471.35	PDK-1	0.07	0.62
G05	PI-103	348.36	PI3K,Autophagy,DNA-PK,mTOR	-0.13	-0.20
G06	Pelitinib (EKB-569)	467.92	EGFR	0.43	0.29
G07	Y-27632 2HCl	320.26	Autophagy,ROCK	-0.14	-0.01
G08	GSK429286A	432.37	ROCK	-0.10	-0.35
G09	ZSTK474	417.41	PI3K	-0.12	-0.21
G10	CCT129202	497.02	Aurora Kinase	-0.21	2.46
G11	Refametinib (RDEA119, Bay 86-9766)	572.34	MEK	-0.04	-0.42
G12	R406 (free base)	470.45	Syk	-0.30	-0.44
G13	ZM 447439	513.59	Aurora Kinase	0.81	0.03
G14	KU-60019	547.67	ATM/ATR	-0.29	-0.34
G15	GSK690693	425.48	Akt	-0.15	-0.42
G16	PF-573228	491.49	FAK	-0.03	1.40
G17	Alisertib (MLN8237)	518.92	Aurora Kinase	0.71	-0.62
G18	AST-1306	621.08	EGFR	#N/D	#N/D
G19	SNS-314 Mesylate	527.04	Aurora Kinase	0.82	0.18
G20	PIK-293	397.43	PI3K	-0.59	-0.13
G21	Regorafenib (BAY 73-4506)	482.82	c-RET,VEGFR	#N/D	#N/D
G22	PIK-294	489.53	PI3K	-0.25	-0.40
G23	GNE-7915	443.4	LRRK2	-0.15	-0.04
G24	Butein	272.25	EGFR	-1.67	1.23
H01	Bosutinib (SKI-606)	530.45	Src	0.13	0.25
H02	AG-1024	305.17	IGF-1R	1.26	0.90
H03	Imatinib Mesylate (STI571)	589.71	c-Kit,Bcr-Abl,PDGFR	-0.31	-0.18
H04	Genistein	270.24	Topoisomerase,EGFR	#N/D	0.79
H05	Rapamycin (Sirolimus)	914.18	Autophagy,mTOR	-0.31	-0.86
H06	Aurora A Inhibitor I	588.07	Aurora Kinase	-0.06	-0.29
H07	Enzastaurin (LY317615)	515.61	PKC	-0.64	-0.75
H08	Pimasertib (AS-703026)	431.20	MEK	-0.18	-0.40
H09	SB216763	371.22	GSK-3	-0.69	-0.86
H10	SAR245409 (XL765)	599.66	PI3K,mTOR	-0.32	-0.79

H11	OSI-906 (Linsitinib)	421.49	IGF-1R	-0.25	-0.64
H12	CP-673451	417.50	PDGFR	0.04	-0.12
H13	GDC-0879	334.37	Raf	-0.14	-0.16
H14	BS-181 HCl	416.99	CDK	-0.15	0.38
H15	JNJ-38877605	377.35	c-Met	-0.47	-0.27
H16	BMS-265246	345.34	CDK	-0.16	-0.47
H17	AT9283	381.43	JAK,Aurora Kinase,Bcr-Abl	0.07	-0.18
H18	AZD8931 (Sapitinib)	473.93	HER2,EGFR	-0.74	-0.22
H19	Lenvatinib (E7080)	426.85	VEGFR	-0.33	-0.21
H20	AZ 960	354.36	JAK	-0.45	0.09
H21	WZ8040	481.01	EGFR	-0.62	-0.16
H22	Telatinib	409.83	VEGFR,PDGFR,c-Kit	0.97	-0.41
H23	VE-821	368.41	ATM/ATR	-0.42	-0.09
H24	Staurosporine	466.53	multiple	2.89	1.55
I01	Volasertib (BI 6727)	618.81	PLK	-0.03	-0.22
I02	WHI-P154	376.20	JAK,EGFR	0.81	0.90
I03	Chrysophanic Acid	254.24	mTOR,EGFR	-0.36	0.01
I04	GNF-2	374.32	Bcr-Abl	-3.26	0.74
I05	LY2603618	436.30	Chk	-0.19	-0.18
I06	Pazopanib	437.52	PDGFR,c-Kit,VEGFR	-0.46	0.49
I07	AS-252424	305.28	PI3K	3.29	0.89
I08	AP26113	529.01	ALK	-0.16	-0.14
I09	NVP-BSK805 2HCl	563.47	JAK	-0.19	-0.35
I10	CX-6258 HCl	498.4	Pim	-0.46	0.05
I11	GSK1838705A	532.57	IGF-1R,ALK	-0.18	0.29
I12	IPA-3	350.45	PAK	-5.37	-12.98
I13	Crenolanib (CP-868596)	443.54	PDGFR	-0.49	0.15
I14	AZD1080	334.37	GSK-3	-0.20	0.14
I15	BGT226 (NVP-BGT226)	650.60	PI3K,mTOR	0.87	0.63
I16	RKI-1447	326.37	ROCK	-0.45	-0.28
I17	Dinaciclib (SCH727965)	396.49	CDK	-0.11	0.09
I18	Ro3280	543.61	PLK	-0.42	-0.05
I19	WP1066	356.22	JAK	-0.31	-2.96
I20	ZCL278	584.89	Rac	0.98	-0.05
I21	Torin 2	432.40	ATM/ATR,mTOR	0.66	0.57
I22	GNE-0877	339.32	LRRK2	-0.29	0.27
I23	WH-4-023	568.67	Src	-0.75	-0.06
I24	GDC-0349	452.55	mTOR	-0.21	0.10
J01	Palomid 529 (P529)	406.43	mTOR	#N/D	-0.46
J02	TG100713	254.25	PI3K	0.46	0.12
J03	Imatinib (STI571)	493.60	PDGFR	-0.26	0.06
J04	S-Ruxolitinib (INCB018424)	306.37	JAK	-0.19	1.11
J05	DCC-2036 (Rebastinib)	553.59	Bcr-Abl	-0.33	-0.30
J06	Piceatannol	244.24	Syk	0.53	-0.36
J07	PF-00562271	665.66	FAK	-0.12	1.82
J08	MEK162 (ARRY-162, ARRY-438162)	441.23	MEK	-0.04	-0.09

J09	R547	441.45	CDK	-0.24	0.15
J10	AZ20	412.51	ATM/ATR	-0.33	-0.25
J11	TAK-901	504.64	Aurora Kinase	-0.19	0.31
J12	PF-3758309	490.62	PAK	0.46	1.58
J13	MK-8776 (SCH 900776)	376.25	CDK,Chk	-0.07	0.02
J14	10058-F4	249.35	c-Myc	-0.10	-0.35
J15	Milciclib (PHA-848125)	460.57	CDK	-0.38	0.48
J16	BIO	356.17	GSK-3	0.32	0.99
J17	Dovitinib (TKI-258) Dilactic Acid	572.59	PDGFR,FGFR,c-Kit,FLT3,VEGFR	-0.18	0.90
J18	AZD2858	453.52	GSK-3	-0.17	-0.01
J19	AZD4547	463.57	FGFR	-0.38	-0.11
J20	AZD9291	499.61	EGFR	-0.51	-0.09
J21	TAE226 (NVP-TAE226)	468.94	FAK	-0.55	0.12
J22	GNE-9605	449.83	LRRK2	-0.32	0.78
J23	AG-18	186.17	EGFR	-0.64	0.24
K01	Degrasyn (WP1130)	384.27	DUB,Bcr-Abl	-0.33	-5.10
K02	GW5074	520.94	Raf	#N/D	-0.94
K03	Phenformin HCl	241.72	AMPK	-0.25	0.03
K04	PF-477736	419.48	Chk	-0.28	0.09
K05	CCT128930	341.84	Akt	0.02	0.88
K06	Cabozantinib malate (XL184)	635.59	VEGFR	#N/D	0.50
K07	Trametinib (GSK1120212)	615.39	MEK	-0.50	-0.56
K08	PP2	301.77	Src	-0.19	-0.15
K09	WAY-600	494.59	mTOR	-0.32	-0.19
K10	CGI1746	579.69	BTK	-0.09	-0.64
K11	AMG-900	503.58	Aurora Kinase	-1.72	-0.36
K12	VE-822	463.55	ATM/ATR	0.33	0.05
K13	TG101348 (SAR302503)	524.68	JAK	-0.47	0.06
K14	LY2835219	602.70	CDK	-0.24	-0.25
K15	HER2-Inhibitor-1	569.63	HER2,EGFR	-0.16	0.87
K16	CNX-2006	545.53	EGFR	-0.33	-0.18
K17	MK-5108 (VX-689)	461.94	Aurora Kinase	-0.06	0.81
K18	NMS-P937 (NMS1286937)	532.52	PLK	-0.15	-0.20
K19	CEP-33779	462.57	JAK	-0.38	-0.16
K20	WZ4003	496.99	AMPK	-0.56	-0.13
K21	TPCA-1	279.29	IκB/IKK	0.88	0.26
K22	Sorafenib	464.82	Raf	#N/D	#N/D
K23	IM-12	377.41	GSK-3	-0.57	-0.12
K24	BMS-345541	255.32	IκB/IKK	-0.22	0.52
L01	BKM120 (NVP-BKM120, Buparlisib)	410.39	PI3K	-0.49	0.34
L02	IKK-16 (IKK Inhibitor VII)	483.63	IκB/IKK	-0.28	-0.30
L03	TAK-733	504.23	MEK	-0.38	-0.09
L04	Go 6983	442.51	PKC	-0.38	0.08
L05	A66	393.53	PI3K	-0.18	-0.23
L06	JNK-IN-8	507.59	JNK	-0.31	-0.37
L07	Flavopiridol HCl	438.30	CDK	1.42	3.52

L08	VS-5584 (SB2343)	354.41	PI3K	-0.19	-0.25
L09	TG101209	509.67	JAK,FLT3,c-RET	-0.64	0.33
L10	PP1	281.36	Src	0.11	0.56
L11	ZM 336372	389.45	Raf	-0.06	0.15
L12	AZD1208	379.48	Pim	-0.20	0.04
L13	PHA-767491	213.24	CDK	-0.03	0.17
L14	SSR128129E	346.31	FGFR	-0.30	0.00
L15	Varlitinib	466.94	EGFR	-0.23	-0.34
L16	Ro 31-8220 Mesylate	553.65	PKC	0.40	1.12
L17	MK-2461	495.55	c-Met,PDGFR,FGFR	-0.22	0.21
L18	CNX-774	499.50	BTK	1.04	0.06
L19	Dabrafenib (GSK2118436)	519.56	Raf	-0.41	-0.13
L20	EHop-016	430.55	Rac	-0.49	0.15
L21	SAR131675	358.39	VEGFR	-0.31	-0.02
L22	KN-62	721.84	Others	-0.92	-1.17
L23	CEP-32496	517.46	CSF-1R,Raf	#N/D	-0.21
M01	Asiatic Acid	488.70	p38 MAPK	-0.23	-0.50
M02	PF-562271	507.49	FAK	-0.08	2.00
M03	AZD5438	371.46	CDK	-0.01	0.30
M04	BAY 11-7082	207.25	IκB/IKK,E2 conjugating	1.02	-2.33
M05	NU7441 (KU-57788)	413.49	DNA-PK,PI3K	-0.66	-1.11
M06	SC-514	224.30	IκB/IKK	-0.55	-0.33
M07	Ibrutinib (PCI-32765)	440.50	BTK	-0.48	-0.62
M08	CZC24832	364.40	PI3K	-0.04	-0.20
M09	GDC-0980 (RG7422)	498.60	mTOR,PI3K	-0.16	-0.12
M10	MK-8745	431.91	Aurora Kinase	-0.10	0.23
M11	PH-797804	477.30	p38 MAPK	-0.18	-0.43
M12	AZD3463	448.95	ALK	-0.39	0.06
M13	PF-04691502	425.48	Akt,mTOR,PI3K	-0.23	-0.15
M14	AVL-292	423.44	BTK	-0.19	-0.33
M15	Wortmannin	428.43	Autophagy,ATM/ATR,PI3K	-0.40	-0.48
M16	GF109203X	412.48	PKC	-0.79	-0.11
M17	AZD2014	462.54	mTOR	-0.33	-0.34
M18	PD173955	443.35	BCR-ABL	#N/D	-1.32
M19	GDC-0068	458.00	Akt	-0.33	-0.31
M20	TG003	249.33	CDK	0.18	-0.32
M21	BI-D1870	391.42	S6 Kinase	5.49	4.28
M22	KN-93 Phosphate	599.03	Others	-0.31	-0.57
M23	Filgotinib (GLPG0634)	425.5	JAK	-0.28	-0.49
M24	ETP-46464	470.52	mTOR,ATM/ATR	-0.49	0.20
N01	Honokiol	266.33	MEK,Akt	0.17	-0.36
N02	Tyrphostin 9	282.38	EGFR	0.32	-0.49
N03	PP121	319.36	DNA-PK,PDGFR,mTOR	2.55	2.05
N04	Icotinib	391.42	EGFR	-0.07	-0.40
N05	GSK2126458 (GSK458)	505.50	PI3K,mTOR	-0.39	-0.34
N06	Tofacitinib (CP-690550) Citrate	504.49	JAK	-0.41	-0.11

N07	AS-604850	285.22	PI3K	-0.22	-0.84
N08	IPI-145 (INK1197)	416.86	PI3K	0.16	-0.47
N09	A-769662	360.39	AMPK	-0.40	-0.79
N10	LDK378	558.14	ALK	-0.14	-0.81
N11	Dacomitinib (PF299804, PF299)	469.94	EGFR	-0.07	-0.26
N12	NU6027	251.28	CDK	-0.11	-0.06
N13	CCT137690	551.48	Aurora Kinase	-0.14	-0.39
N14	SKI II	302.78	S1P Receptor	-2.32	-0.06
N15	CUDC-907	508.55	PI3K,HDAC	0.10	-0.49
N16	GSK650394	382.45	Others	-0.34	-2.40
N17	TAK-285	547.96	EGFR,HER2	1.54	0.57
N18	CO-1686 (AVL-301)	555.55	EGFR	-0.24	-0.16
N19	INK 128 (MLN0128)	309.33	mTOR	0.16	-0.10
N20	6H05	590.14	Rho	-0.18	-0.61
N21	Semaxanib (SU5416)	238.28	VEGFR	-0.35	1.52
N22	AR-A014418	308.31	GSK-3	-0.59	-0.82
N23	AZD5363	428.92	Akt	-0.13	0.54
N24	GW297361X	372.417	VRK1	3.89	3.29
O01	Indirubin	262.26	GSK-3	-1.00	-0.14
O02	ZM 323881 HCl	411.86	VEGFR	#N/D	-0.32
O03	OSI-027	406.44	mTOR	-0.14	-0.20
O04	CHIR-99021 (CT99021) HCl	501.80	GSK-3	-0.17	-0.14
O05	WYE-125132 (WYE-132)	519.60	mTOR	-0.10	-0.27
O06	Fingolimod (FTY720) HCl	343.90	S1P Receptor	-0.20	-0.50
O07	CAY10505	289.28	PI3K	-0.37	-0.64
O08	XL019	444.53	JAK	-0.07	-0.26
O09	CH5132799	377.42	mTOR,PI3K	-0.07	-0.21
O10	GSK2334470	462.59	PDK-1	-0.33	-0.26
O11	AG-1478 (Tyrphostin AG-1478)	315.75	EGFR	-0.03	0.40
O12	TIC10	386.49	Akt	-0.01	-0.31
O13	AZ 628	451.52	Raf	-0.43	-0.25
O14	PF-543	465.6	S1P Receptor	0.00	-0.72
O15	NVP-BVU972	340.38	c-Met	-0.62	-0.28
O16	Skepinone-L	425.42	p38 MAPK	-0.07	-0.92
O17	Tofacitinib (CP-690550,Tasocitinib)	312.37	JAK	-1.15	0.17
O18	PFK15	260.29	Others	3.30	-5.11
O19	BYL719	441.47	PI3K	-0.80	-0.50
O20	K-Ras(G12C) inhibitor 9	513.78	Rho	-0.17	-0.59
O21	Golvatinib (E7050)	633.69	VEGFR,c-Met	-0.80	-0.52
O22	LDC000067	370.43	CDK	1.40	0.10
O23	GSK2636771	433.42	PI3K	-0.85	-0.10
O24	Pacritinib (SB1518)	472.58	JAK	-0.04	0.11
P01	Quercetin	302.24	PKC,Src,PI3K,Sirtuin	1.12	-0.14
P02	ZM 306416	333.74	VEGFR	-0.02	0.97
P03	Fostamatinib (R788)	580.46	Syk	-0.07	-0.47
P04	TAK-715	399.51	p38 MAPK	-0.19	-0.02

P05	A-674563	358.44	PKA,CDK,Akt	1.23	1.73
P06	VX-702	404.30	p38 MAPK	0.14	-0.22
P07	CHIR-124	419.91	Chk	0.11	2.68
P08	PD168393	369.22	EGFR	0.27	-0.32
P09	KX2-391	431.53	Src	-0.01	-0.55
P10	Zotarolimus(ABT-578)	966.21	mTOR	-0.27	-0.96
P11	SB415286	359.72	GSK-3	0.02	-0.11
P12	CGK 733	555.84	ATM/ATR	-0.55	0.23
P13	AMG-458	539.58	c-Met	-0.11	-0.34
P14	GZD824	724.77	Bcr-Abl	-0.16	-1.21
P15	3-Methyladenine	149.15	Autophagy,PI3K	0.00	-0.07
P16	Losmapimod (GW856553X)	383.46	p38 MAPK	-0.26	-0.32
P17	Sotrastaurin	438.48	PKC	0.59	0.56
P18	TAK-632	554.52	Raf	-0.50	#N/D
P19	Tyrphostin AG 879	316.46	HER2	0.66	0.28
P20	HS-173	422.46	PI3K	0.48	0.26
P21	IMD 0354	383.67	IκB/IKK	0.28	-0.61
P22	EHT 1864	581.47	Rho	-0.44	0.07
P23	TCS 359	360.43	FLT3	0.49	-0.22
P24	DMSO	78.13	reference	0.09	0.10
